# Myths about diabetes and its treatment in North Indian population

**DOI:** 10.4103/0973-3930.54290

**Published:** 2009

**Authors:** Mridula Rai, Jugal Kishore

**Affiliations:** Department of Community Medicine, Maulana Azad Medical College, New Delhi, India

**Keywords:** Diabetes, Myths, North India, treatment

## Abstract

**BACKGROUND:**

Myths prevailing about diabetes in the society have become a major hurdle for its proper treatment and control.

**AIM:**

To find out about various myths related to diabetes and its treatment in the population.

**MATERIALS AND METHODS:**

A cross sectional study was carried out in a teaching hospital of Delhi in 2008. 124 diabetic patients attending the regular diabetic clinic, 78 people who accompanied these patients and 214 non-diabetic people were included in the study. A pre-tested interview schedule with 48 questions was used to get information about sociodemographic characteristics and myths about diabetes. Data was analyzed by Epi info software version 3.2.

**RESULTS:**

The most common myth in the population (22%) was that eating more sugar causes diabetes. Others were: diabetes can only occur in old age, soaking feet in water can help control blood sugar, diabetes is a result of past sins and is cured by spiritual treatment. Myths were significantly more common in females, non-diabetics, less educated group. There was a slightly higher prevalence of myths in Muslim population. 12.1% of diabetics were taking herbal medicines. 15.9% of the diabetics and 26% of non diabetics were unaware that complications could occur if diabetes was uncontrolled. 18.5% of diabetics and 30.1% of non-diabetics were unaware about role of diet and lifestyle measures in control of diabetes.

**CONCLUSIONS:**

The prevalence of myths about diabetes is high in North Indian population which could be associated with poor early health seeking behavior and poor compliance with treatment.

## Introduction

Diabetes, with its attendant acute and long term complications, and the myriad of disorders associated with it, is a major health hazard. In keeping with scenarios of most developing countries, India has long passed the stage of diabetes epidemic. The problem has now reached pandemic proportions. It is a large public health problem growing astronomically every year. Apart from treatment of diabetes we need to pay attention to the prevention and health education of people about the disease.

Myths are defined as stories shared by a group of people which are a part of their cultural identity. They have a strong influence in the life of individuals and their way of living including seeking treatment during illness. Therefore, understanding the myths and misconceptions about the disease, like diabetes mellitus, is important in providing excellent care and health education to both patients and healthy individuals.[1] Indian population consists of people from different cultural backgrounds and there is a very strong influence of the various myths on health seeking behavior in our population. People believe in spiritual treatment and alternative forms of medicine, instead of coming to a doctor they visit a hakim (local traditional practitioner). There have been emergencies reported in cases of diabetes mellitus where the patients delayed their presentation to the doctor due to these myths.

There has been little research to understand these myths. Not many studies have been done and we do not have much data related to this subject. Therefore, we have tried to find out the prevalence of common myths about diabetes and its treatment and to correlate them with the peoples% sociodemographic characteristics.

## Materials and Methods

We conducted a cross sectional study in a tertiary care hospital of Delhi in 2008 to assess the prevalence of myths related to diabetes and its treatment. The hospital caters to a tremendous load of patients representing the entire north India.

The subjects of the study included:

1. 124 diabetic patients attending the weekly diabetic clinic at the hospital.
2. The non-diabetic population consisted of- the relatives accompanying the patients attending the diabetic clinic. Of 124 patients, 78 were accompanied by relatives, and were included in the study. Also 118 consecutive non-diabetic patients attending general outpatient clinics and the relatives and friends accompanying these patients (96) representing the healthy non-diabetic population were included.
  Each study subject was explained the aims and protocol of the study and was asked for consent to participate. Of a total of 429 persons approached, 416 (96.9%) agreed to participate. A pretested semistructured interview schedule containing 48 questions regarding sociodemographic profile, knowledge and myths and about diabetes and its treatment was used to get information from the study group. The interview schedule was prepared in Hindi (the local language) and tested on separate group of patients and validated with the help of language and medical experts. The interview schedule was administered by the first author in diabetic and general outpatient clinic. Each interview took an average of 15 minutes. Only after completion of data collection from one subject was the next subject enrolled. All the study subjects were new patients to avoid repetition. Each filled interview schedule was rechecked for any missing information. The data was collected over a period of 2 months and was analyzed with the help of Epi-info computer software package of the WHO (version 3.2).

## Results

The study group consisted of 124 diabetic patients attending the regular diabetic clinic, 78 people who accompanied these patients and 214 non-diabetic people. The sample consisted of 201 males (48.3%) and 215 females (51.7%) [Table 1].

**Table 1:** Demographic profile of study population

	n= 416
Diabetic/ Non diabetic	124/ 292
Males/ Females	201/ 215
Age <35	21
35-55	194
>55	209
Education <Class V	81
Class V- Class X	179
>Class X	156
Diabetes	
No Treatment	88
On drugs	33
On Insulin	3

The most common myth in the population (22%) was that eating more sugar causes diabetes. Others were- diabetes can only occur in old age (7.2%), soaking feet in water can help control blood sugar (11.8%), diabetes is a result of past sins and it can be cured by spiritual treatment (9.4%) [Table 2].

**Table 2:** Prevalence of myths in the study population

	n= 416
Diabetes occurs because of increased sugar intake	92 (22.1)
Soaking feet in water helps decrease blood sugar level	49 (11.8)
Diabetes occurs because of past sins and can be cured by spiritual treatment	39 (9.4)
Diabetes can be cured by herbal treatment	75 (18.0)
There is no role of diet/ lifestyle measures in cure of diabetes	111 (26.7)
Normal blood sugar level <200	266 (63.9)
Once on drugs and insulin you can have anything	86 (20.7)
Diabetes occurs only in old age	30 (7.2)
Diabetes is contagious	74 (17.8)
No complications can occur even if diabetes is uncontrolled	96 (23.1)

Figures in parentheses are in percentage

We compared the prevalence of myths in diabetics and non-diabetics, Hindus and Muslims, males and females and according to the educational status of the study population. The findings are summarized in Table 3–6.

**Table 3:** Comparison of myths in Diabetics and nondiabetics

	Diabetics (n= 124)	Non Diabetics (n= 292)	*P*
High sugar intake causes diabetes (92)	18 (14.5)	74 (25.3)	0.01*
Diabetes is contagious (74)	5 (4.0)	69 (23.6)	< 0.01*
Diabetes occurs in old only (30)	8 (6.4)	22 (7.5)	0.69
Belief in cure by herbal medicine (75)	15 (12.1)	60 (20.5)	0.03*
Belief in cure of diabetes by spiritual treatment (39)	11 (8.9)	28 (9.6)	0.81
No role of lifestyle changes in diabetes (111)	23 (18.5)	88 (30.1)	0.01*

Figures in parentheses are in percentage

The findings [Table 3] suggest that prevalence of myths was higher in non diabetics. The variables that showed significant association were high sugar intake causes diabetes (*P*=0.01), diabetes is contagious (*P*<0.01), herbal medicine is the cure for diabetes (*P*=0.03), there is no role of lifestyle changes in treatment of diabetes (*P*=0.01). This can be explained because the diabetics included in our study were those who were visiting clinics and were in touch with healthcare professional, getting education from them about their disease. On the other hand the non-diabetics were not receiving any such education.

Table 4 describes the comparison of males and females in reporting myths about diabetes mellitus. The females reported myths more as compared to males. The variables that showed significant association with female gender, were increased intake of sugar causes diabetes (*P*=0.02), diabetes occurs only in old people (*P*=0.01) and it can be cured by spiritual treatment (*P*=0.008). The other variables did not show any significant association.

**Table 4:** Comparison of myths with respect to gender

	Males (201)	Females (215)	*P*
High sugar intake causes diabetes (92)	35 (17.4)	57 (26.5)	0.02*
Diabetes is contagious (74)	33 (16.4)	41 (19.1)	0.48
Diabetes occurs in old only (30)	8 (4.0)	22 (10.2)	0.01*
Belief in cure by herbal medicine (75)	43 (21.4)	32 (14.9)	0.08*
Belief in cure of diabetes by spiritual treatment (39)	11 (5.5)	28 (13.0)	0.008*
No role of lifestyle changes in diabetes (111)	48 (23.9)	63 (29.3)	0.21

Figures in parentheses are in percentage

Prevalence of myths was compared according to the educational status, they were more prevalent in less educated group reaching significant level in all the variables except diabetes can be cured by herbal medicines- eating more sugar causes diabetes (*P*=0.04), it occurs only in old age (*P*=0.02), diabetes is contagious (*P*<0.01), belief in spiritual cure (*P*=0.005) and there is no role of lifestyle measures in its treatment (*P*=0.03) [Table 5].

**Table 5:** Association of myths with the educational status

	< Class V (81)	Class V- X (179)	> Class X (156)	*P*
High sugar intake causes diabetes (92)	24 (29.6)	32 (17.9)	26 (16.7)	0.04*
Diabetes is contagious (74)	33 (40.7)	24 (13.4)	17 (10.9)	<0.01*
Diabetes occurs in old only (30)	11 (13.6)	13 (7.3)	6 (3.8)	0.02*
Belief in cure by herbal medicine (75)	13 (16.0)	34 (19.0)	28 (17.9)	0.84
Belief in cure of diabetes by spiritual treatment (39)	15 (18.5)	15 (8.4)	9 (5.8)	0.005*
No role of lifestyle changes in diabetes (111)	31 (38.3)	43 (24.0)	37 (23.7)	0.03*

Figures in parentheses are in percentage

When myths were compared according to the religion most of the variables had similar prevalence in both Muslims and Hindus except for the belief in cure of diabetes by spiritual treatment which was significantly higher in Muslims (*P*=0.02) [Table 6].

**Table 6:** Comparison of prevalence of myths according to religion

	Hindu (264)	Muslim (152)	*P*
High sugar intake causes diabetes (92)	58 (22.0)	34 (22.4)	0.92
Diabetes is contagious (74)	48 (18.2)	26 (17.1)	0.78
Diabetes occurs in old only (30)	17 (6.4)	13 (8.5)	0.42
Belief in cure by herbal medicine (75)	46 (17.4)	28 (18.4)	0.79
Belief in cure of diabetes by spiritual treatment (39)	18 (6.8)	21 (13.8)	0.02*
No role of lifestyle changes in diabetes (111)	66 (25.0)	45 (29.6)	0.31

Figures in parentheses are in percentage

## Discussion

Myths can be prevalent in a population due to a variety of reasons like poor education, cultural beliefs and social misconceptions. They are usually passed on from one generation to the next. It is difficult to break this chain as it is deep seated in the society. We need to change the mindset and the behavior of the population to eliminate the myths and educate the people about Diabetes.

It is important to know about these myths and misconceptions prevalent in the population as understanding them is essential to provide good care as well as health education to the people.

We tried to focus on the common myths prevalent in North India. The most widely believed myth was that eating more sugar causes diabetes. This is not entirely true as it is not directly related to eating sugar, but is very much affected by diet in general. Some people also believe that soaking feet in water helps in decreasing blood sugar level which is entirely incorrect.

There is also a group of people who associate the presence of diabetes to past sins and think that spiritual treatment can cure it. Some others are of opinion that herbal medicines are very effective in treatment of diabetes. These sections of people often present late to Doctors with complications as they first seek spiritual or herbal treatment. Nisar *et al*, reported high prevalence of such beliefs in spiritual treatment in Karachi, Pakistan.[2] Another study conducted in Pakistan, reported similar findings that patients abandoned traditional medicine when their serum glucose soared.[3] Performing hawans, pujas and going to faith healers for treatment is common in chronic diseases like diabetes, cancer and mental illnesses.[4,5]

People had a misconception that diabetes can occur only in old age. This is not true and they need to be made aware that in regard to age, diabetes spares no age group. There is certainly a higher chance of developing type 2 diabetes as you get older but people of all ages, including children can get diabetes.

Another myth that surrounds diabetes is that it is contagious. This was also found in the study done in Pakistan by Nisar *et al*.[2] Also people are not aware about the normal blood sugar levels, role of lifestyle changes in control and treatment of diabetes and that if not treated diabetes can lead to complications.

The prevalence of myths was found to be higher in females. This is contrary to what was found in the study by Nisar *et al* in Pakistan.[2] The higher incidence of myths in females can be attributed to the fact that females are usually less educated than males in India.

Educational status of people seemed to reduce their belief in the myths and they were better informed about the disease. This is similar to what was found by Nisar *et al*.[2]

This study therefore clearly reflects that prevalence of myths and misconceptions about diabetes and its treatment is high in North India and this could be a major hindrance in control and prevention of diabetes which is a disease of national importance.

## Conclusion

Myths and misconceptions about diabetes can hinder seeking of treatment by people. In this study education has been shown to be associated with decreased belief in myths and more knowledge about the disease. We need to educate people about this disease and its treatment options. This will play a very important role in control and prevention of diabetes in the country.
